# 

**Published:** 1855-04

**Authors:** 


### CONTENTS

ORIGINAL COMMUNICATIONS:— PAGE

ART. I.—Notes of Experiments on Ulcers and Wounds. By Daniel Brainard, M.D., . . . . . . . . . . . . 145

ART. II.—Nitrate of Silver as a Local Remedy in Phlegmasia Dolens. By J. R. Mills M.D., . . . . . . . . . . 150

ART. III.—Chloroform; its Uses, etc. By P. A. Allaire, M.D., . . 151

ART. IV.—Cases from the Note Book. By Owen Long, M.D., . 159

SELECTIONS:

A New Broth for the Sick, . . . . . . . . . . 164

Uses of the Microscope in the Diagnosis of Cancer, . . . 165

A Case of Vertebral Dislocation, with Favorable Termination. . . 167

Indications for Treatment in Fever afforded by the State of the Heart, 169

BOOK NOTICES, . . . . . . . . . . 171

EDITORIAL:

The American Medical Association, . . . . . . . 180

Convention of Delegates from Medical Colleges, . . . . 181

Cook County Medical Society, . . . . . . . . . 182

Decrease of Medical Students, . . . . . . . . 186

Miscellaneous, - - - - - - - - - - - 188

